# Identification, functional prediction, and key lncRNA verification of cold stress-related lncRNAs in rats liver

**DOI:** 10.1038/s41598-020-57451-7

**Published:** 2020-01-16

**Authors:** Hong Ji, Chunyang Niu, Xuelong Zhan, Jing Xu, Shuai Lian, Bin Xu, Jingru Guo, Li Zhen, Huanmin Yang, Shize Li, Li Ma

**Affiliations:** College of Animal Science and Veterinary Medicine, Heilongjiang Bayi Agricultural University, Daqing, 163319 China

**Keywords:** High-throughput screening, High-throughput screening, High-throughput screening, Gene expression, Long non-coding RNAs

## Abstract

Cold stimulation reduces the quality of animal products and increases animal mortality, causing huge losses to the livestock industry in cold regions. Long non-coding RNAs (lncRNAs) take part in many biological processes through transcriptional regulation, intracellular material transport, and chromosome remodeling. Although cold stress-related lncRNAs have been reported in plants, no research is available on the characteristic and functional analysis of lncRNAs after cold stress in rats. Here, we built a cold stress animal model firstly. Six SPF male Wistar rats were randomly divided to the acute cold stress group (4 °C, 12 h) and the normal group (24 °C, 12 h). lncRNA libraries were constructed by high-throughput sequencing (HTS) using rat livers. 2,120 new lncRNAs and 273 differentially expressed (DE) lncRNAs were identified in low temperature environments. The target genes of DElncRNA were predicted by cis and trans, and then functional and pathway analysis were performed to them. GO and KEGG analysis revealed that lncRNA targets were mainly participated in the regulation of nucleic acid binding, cold stimulation reaction, metabolic process, immune system processes, PI3K-Akt signaling pathway and pathways in cancer. Next, a interaction network between lncRNA and its targets was constructed. To further reveal the mechanism of cold stress, DElncRNA and DEmRNA were extracted to reconstruct a co-expression sub-network. We found the key lncRNA MSTRG.80946.2 in sub-network. Functional analysis of key lncRNA targets showed that targets were significantly enriched in fatty acid metabolism, the PI3K-Akt signaling pathway and pathways in cancer under cold stress. qRT-PCR confirmed the sequencing results. Finally, hub lncRNA MSTRG.80946.2 was characterized, and verified its relationship with related mRNAs by antisense oligonucleotide (ASO) interference and qRT-PCR. Results confirmed the accuracy of our analysis. To sum up, our work was the first to perform detailed characterization and functional analysis of cold stress-related lncRNAs in rats liver. lncRNAs played crucial roles in energy metabolism, growth and development, immunity and reproductive performance in cold stressed rats. The MSTRG.80946.2 was verified by network and experiments to be a key functional lncRNA under cold stress, regulating *ACP1*, *TSPY1* and *Tsn*.

## Introduction

lncRNAs are non-coding RNAs of greater than 200 nt in length. Unlike mRNAs, the primary structure of lncRNAs are not highly conserved, and a considerable number of lncRNAs are transcripted from introns, exons, intergenic regions and overlapping regions^1^. lncRNAs are the scientific frontier of the genome era and may reveal new RNA-mediated genetic expression regulatory networks, which could clearer clarify function of genome from the perspective of non-coding RNAs. lncRNA works mainly in following ways: lncRNA remodels chromatin^2^, DNA binding^3^, promoting chromatin formation^4^, acting effects on RNA processing^5^, affecting mRNA stability^6^ and lncRNA can directly regulate the function of proteins^7^. With the application of high-throughput sequencing, lots of biologically functional lncRNAs have been discovered. In mammals, lncRNAs participate in regulating important physiological processes such as individual neurodevelopment^8^, cell cycle^9^, cell protection^10^, and tumor development and metastasis^11^.

In cold regions, animals are prone to cold stress and their physical development is affected. In pregnant animals, cold stress can result in symptoms such as miscarriage and even infertility. The stress response caused by cold stimulation can cause damage to the nervous, cardiovascular, and immune systems^12,13^. However, reports on the function of cold stress-related lncRNA are rare. Su *et al*. studied overexpression of lncRNA TUG1 (taurine up-regulated gene 1) in mice can prevent cold-induced damage^14^. lncRNAs associated with cold stress have also been reported recently in cabbage and cassava^15,16^. Kidokoro found that soybean (C repeat binding factor) CBF/DREB1 (dehydration response element binding protein 1) regulates gene expression in cold response process^17^. This process activates many defense mechanisms, including molecular chaperones, metabolite biosynthesis enzymes and so on. Freezing and low temperature stress can cause plant metabolic disorder and increase the production of various reactive oxygen species. H_2_O_2_ can regulate gene expression under cold stress, affecting transduction in wild type and catalase (Δ*katG*)/thioredoxin peroxidase (*tpx*) cells treated by cold stress^18^. Keeping body temperature constant under cold environment needs heat production and protection. These mechanisms are affected by various neurotransmitters and hormones, and regulated by the nervous system^19^. Numerous studies have shown that cold stress affects multiple metabolic and molecular regulatory processes *in vivo*. PACAP (Pituitary adenylate cyclase activating polypeptide) acts a pivotal part in peripheral and central physiological stress responses. Cline found that PACAP is involved in thermostimulated sympathetic signaling and may be a crucial regulator of lipid metabolism^20^. Environmental factors such as cold stress may lead to mammal hippocampus apoptosis in late pregnancy, and in a caspase-3-independent manner to enhance phosphorylation of Ser536 by P65^21^. In addition, some lncRNAs can regulate cell function through other pathways. For example, Kang *et al*. found that energy-induced lncRNA HAND2-AS1 (heart and neural derivatives expressed 2-antisense 1) inhibits HIF-1 (hypoxia inducible factor-1) α-mediated energy metabolism and inhibits osteosarcoma development^22^. However, the regulation of lncRNA involvement in cold stress in rat livers remains unclear.

As an important organ of heat production in the body, the liver increases its activity and heat production during acute cold stimulation to maintain the body’s normal temperature^23^. In this process, how lncRNAs play regulatory roles needs further study. Here, we analyzed and identified the characteristics of lncRNAs in liver of cold-stressed rats, predicted the target genes of these differential lncRNA by cis and trans, and explored the roles of lncRNAs under cold stress in rat liver. Our data will help to better understand the mechanisms of lncRNA in rat liver under cold stress.

## Results

### Identification of lncRNAs in liver of rats

**Figure 1:**
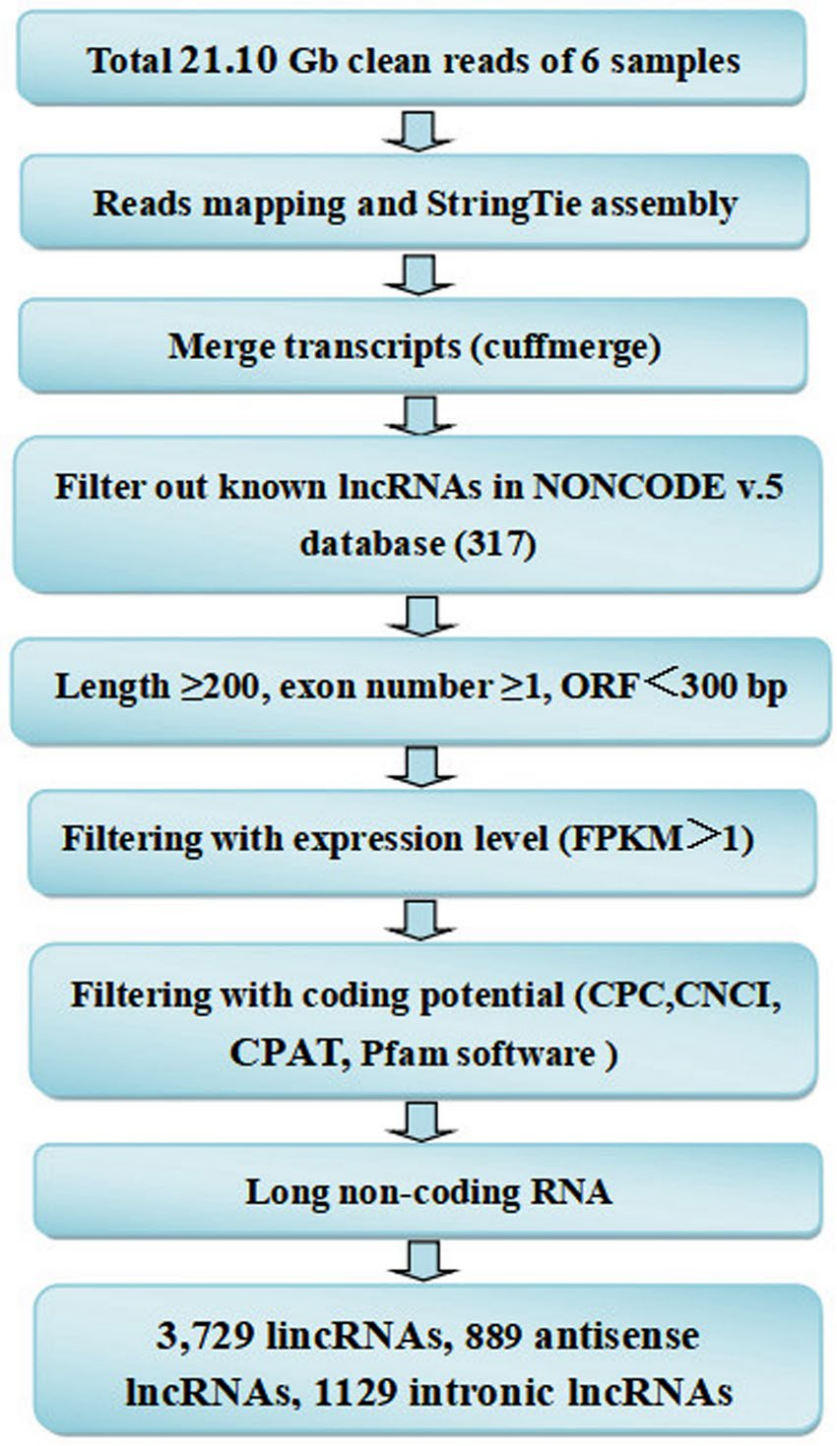
Informatics pipeline for the identification of lncRNAs.

**Figure 2:**
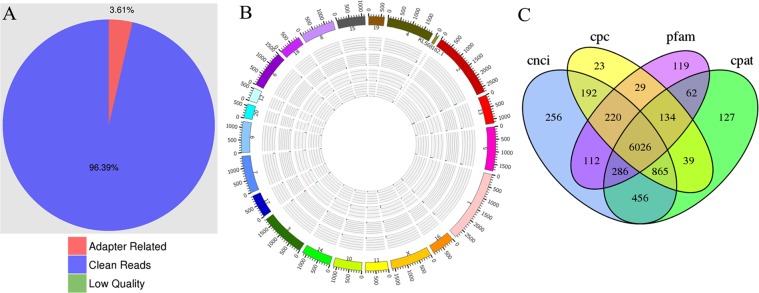
Genomic features of lncRNAs. (**A**) Raw data composition. (**B**) Distribution of lncRNAs on each chromosome. (**C**) Venn diagram to predict the coding ability of lncRNAs.

Six rats were randomly selected respectively in the normal group (L01, L02 and L03) and the stress group (L04, L05 and L06). Then total RNA was extracted from the liver samples and six cDNA libraries were constructed. After quality control of raw data from each sample, high quality data remained nearly 21.10 Gb, accounting for approximately 96.39% of the total. Afterwards, screening of dependable candidate lncRNAs from assembled transcripts based on process pipelines for high-throughput sequencing data (Fig. 1). Clear statistics on the quality of data and the proportion of raw data are shown in Fig. 2A. The distribution of lncRNAs on each chromosome is shown in Fig. 2B. These lncRNAs are evenly distributed on all chromosomes. It is worth noting that the number of lncRNAs on chromosomes 1 and 2 were relatively high, respectively 513 (8.5%) and 304 (5.04%). Four different tools, Coding-Potential Assessment Tool (CPAT), Coding-Non-Coding Index (CNCI), Pfam-scan and Coding Potential Calculator (CPC) were used to calculate the ability of transcripts to encode proteins (Fig. 2C).

### Characterization of cold stress-related lncRNAs

**Figure 3:**
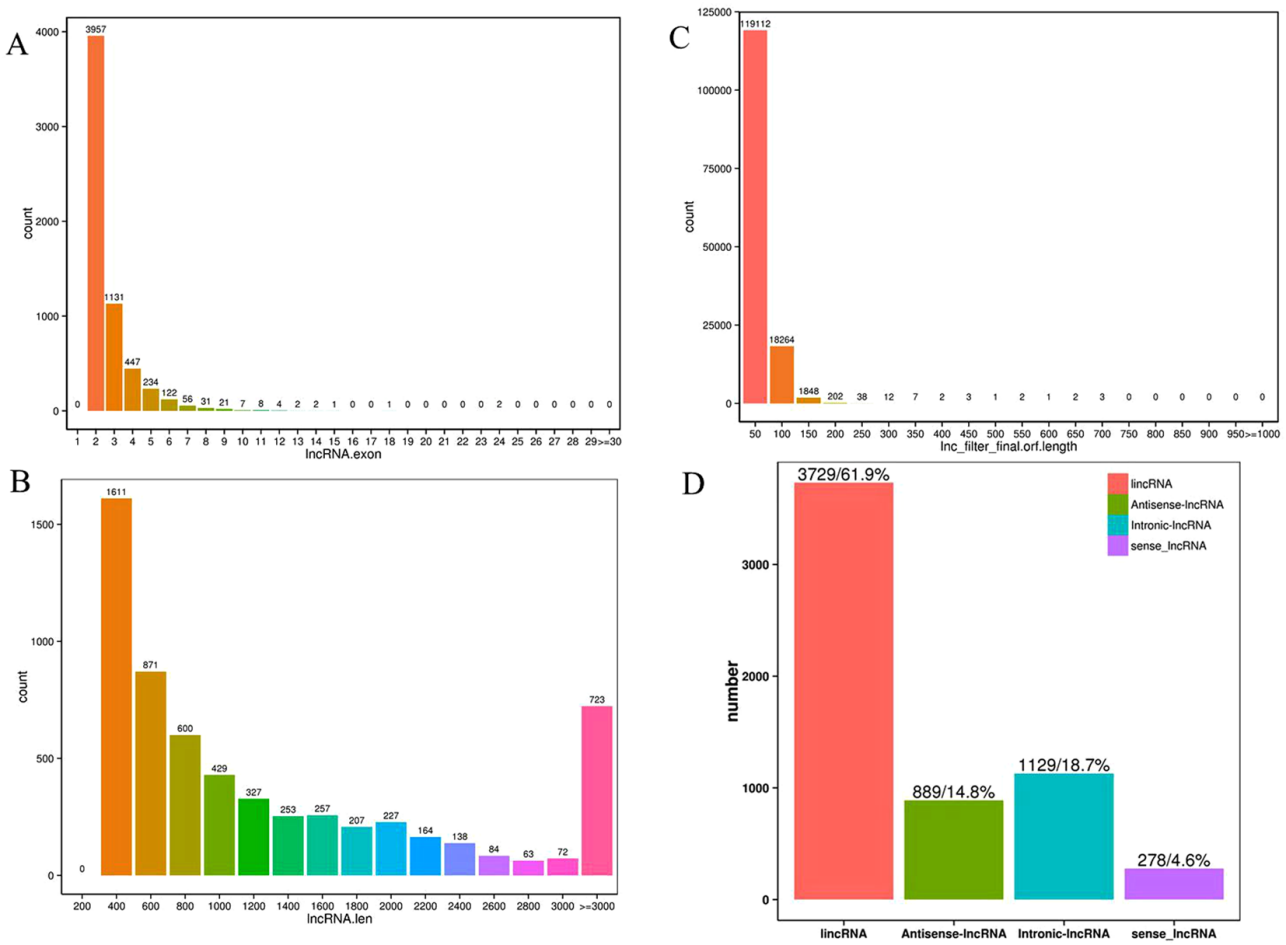
Specific characteristics of rat lncRNAs. (**A**) Rat lncRNAs exon number chart. (**B**) Length distribution of lncRNAs of rat species. (**C**) lncRNAs orf length statistics. (**D**) Proportion of lincRNA, antisense lncRNAs, intronic lncRNAs and sense lncRNAs identified in rat.

We described the genomic characteristics of the acquired cold stress-related lncRNAs in rats liver. A large number of the lncRNAs contained two exons (Fig. 3A). Consistent with the size distribution pattern of the lncRNA library, the length of library lncRNAs at 300–500 nt was most distributed, and the number of lncRNAs for this length was 1,611. There were 723 lncRNAs with tags larger than 3000 nt (Fig. 3B). Most lncRNAs (85%) contained a short ORF (open reading frame) (approximately 20–60 amino acids), which is shorter than for codeRNA (Fig. 3C). From Figs. 3D, 6,025 candidate lncRNAs were captured, including 3,729 lincRNAs (61.9%), 889 antisense lncRNAs (14.8%), 1129 intronic lncRNAs (18.7%) and 278 sense lncRNAs (4.6%). Of these lncRNAs, 317 (5.2%) were identified as known using BLAST alignment with the rat lncRNAs in NONCODEv5 database^24^, and we obtained a grand total of 451 novel lncRNAs.

**Figure 4:**
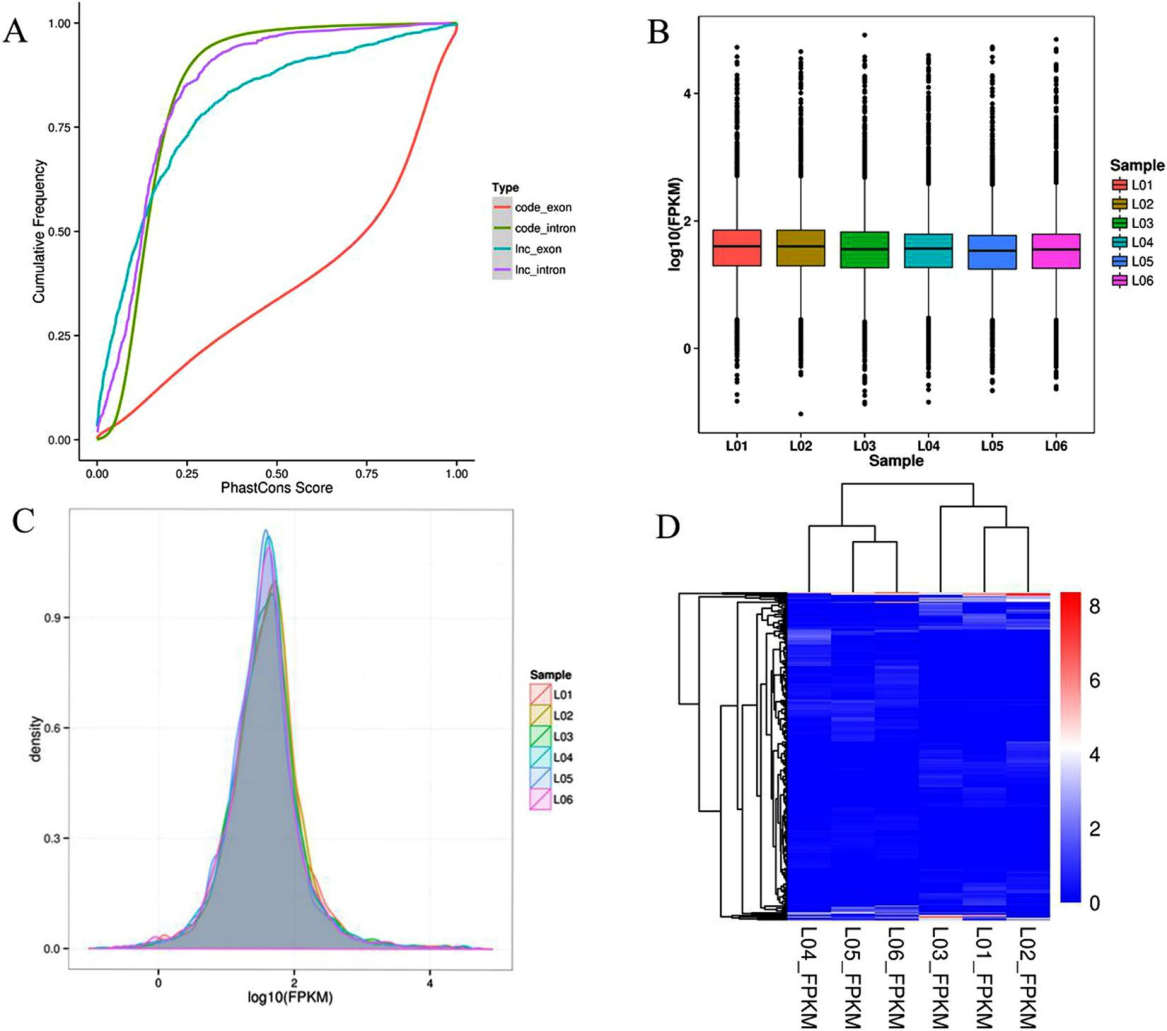
Expression models of rat lncRNAs. (**A**) The cumulative curve of the average phastCons score of lncRNAs-exon (blue), lncRNAs-intron (purple), coding genes (red) and genomic background (green). Genomic background was randomly selected from the whole genome. (Graph legend: L01-L03 FPKM: lncRNAs in the normal group, L04-L06 FPKM: lncRNAs in the cold stress group.) (**B**) The box plot of expression levels of rat lncRNA in the cold stress group and the normal group. The y-axis represents the average log10 (FPKM) value of each set of replicates. (**C**) Comparison of FPKM density distribution between two groups. (**D**) Hierarchical clustering of the diferentially expressed lncRNAs.

We assessed the conservation of rat lncRNAs (Fig. 4A). Most lncRNAs scores ≤0.4, indicating poor conservation. Moreover, the overall distribution of lncRNAs expression was presented by the FPKM (fragments per kilobase million) density distribution comparison chart and the FPKM box plot. By detecting the expression pattern of lncRNAs, it was shown that the expression profiles of the three biological replicates in the cold stress group and the normal group were relatively close, indicating that the experimental repeated data was will (Fig. 4B). The FPKM value of lncRNAs expression levels spanned 10^−2^ to 10^4^ six orders of magnitude (Fig. 4C). In Fig. 4D, we quantified the expression level of the lncRNAs using Stringtie. The clustering plots showed significant differences in lncRNA expression between the groups, but the differences within each group were small.

### Differential expression of lncRNAs

**Figure 5:**
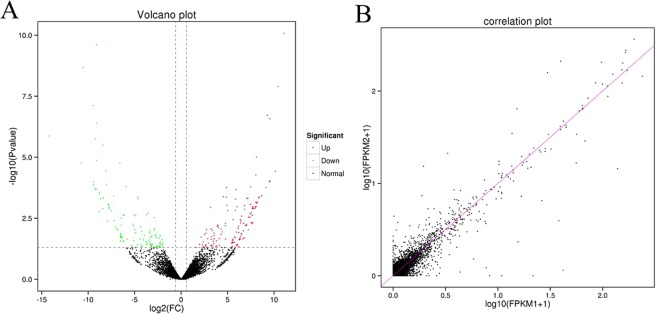
Differentially expressed lncRNAs. (**A**) The Volcano plot of DElncRNAs between the cold stress group and the normal group. Red color represents significant up-regulated and green color represents significant down-regulated. (**B**) The correlation plot, three repeated samples, the points are concentrated in the diagonal, indicating a high correlation coefficient. Correlation was evaluated by Pearson’s correlation coefficient of total lncRNAs expression levels.

Expression levels were analyzed using the ‘ballgown’ R package to screen differential expressed (DE) lncRNAs between the cold stress group and the normal group. We identified 146 up-regulated lncRNAs (53.5%) and 127 down-regulated lncRNAs (46.5%), a total of 273 significant DElncRNAs. The top 20 significant DElncRNAs were demonstrated in Table 1. The volcano plot in Fig. 5A depicted the approximate distribution of DElncRNAs. In our study, 2120 novel lncRNAs were acquired, including 435 antisense lncRNAs, 1618 lincRNAs and 67 intronic lncRNAs. The targets of DElncRNAs were predicted based on trans and cis-acting. Furthermore, the targets of 58 lncRNAs had functional annotations. From the correlation plot, it can be seen that lncRNA expression is highly correlated within the group (Fig. 5B).

**Table 1:** Differentially expressed lncRNAs in liver samples.

lncRNA	P-Value	FDR	log2FC	regulated	length (bp)
**Top 20 differential expressed lncRNAs**					
MSTRG.56167.3	8.44E-11	4.61E-07	11.10088491	up	2858
MSTRG.487.14	2.45E-10	6.70E-07	−9.107494828	down	1414
MSTRG.65671.4	2.10E-09	3.82E-06	−10.51898195	down	258
MSTRG.23974.3	1.26E-08	1.72E-05	10.44064717	up	6389
MSTRG.4553.16	7.73E-08	8.44E-05	−9.459919615	down	3451
MSTRG.65602.6	1.91E-07	0.000174097	9.287886208	up	3182
MSTRG.30092.2	2.66E-07	0.000207222	9.549994454	up	6710
MSTRG.7147.36	4.07E-07	0.000277635	−9.09401165	down	1800
MSTRG.488.3	1.35E-06	0.000819159	−14.19999886	down	1498
MSTRG.7147.38	1.79E-06	0.000979921	−9.204395271	down	1862
MSTRG.55788.4	3.26E-06	0.001618105	−8.406993621	down	1230
MSTRG.81485.14	9.94E-06	0.00452224	8.117395911	up	2558
MSTRG.75398.3	1.36E-05	0.005714814	−9.074906964	down	1327
MSTRG.7146.36	1.73E-05	0.006314205	−10.77186301	down	1897
MSTRG.77439.2	1.73E-05	0.006314205	−6.583252364	down	1015
MSTRG.52429.7	3.65E-05	0.012155208	−8.077264313	down	1380
MSTRG.7146.42	3.78E-05	0.012155208	10.14630706	up	1963
MSTRG.60605.1	5.38E-05	0.016324479	8.051430684	up	6118
MSTRG.25014.4	9.39E-05	0.027004173	9.57666706	up	6097
MSTRG.7147.7	0.000103965	0.0270409	−9.418430487	down	2206

### Functional and pathway analysis of DElncRNA targets

**Figure 6:**
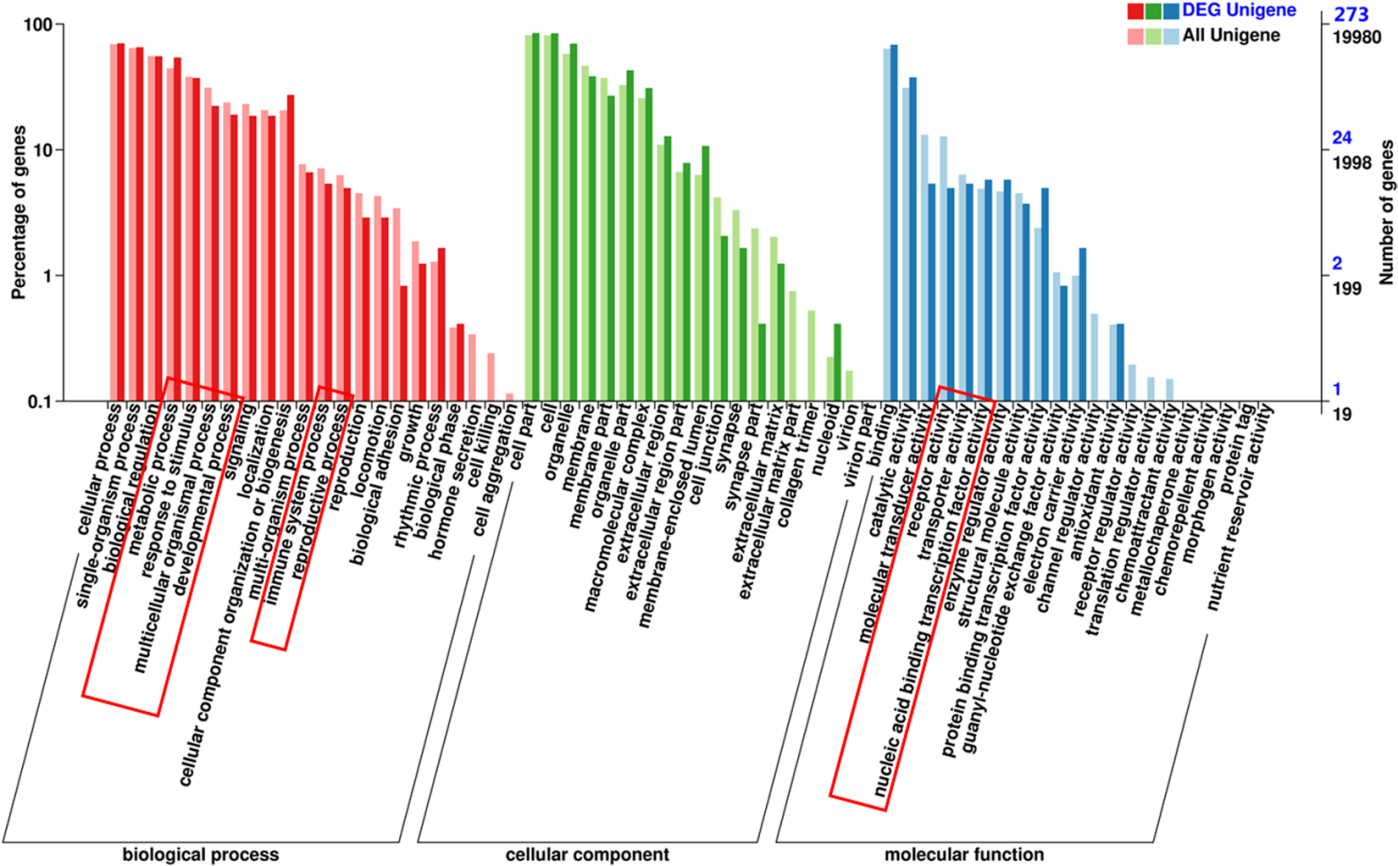
GO classification of target genes of DElncRNAs in rats. The abscissa was the GO classification, the left side of the ordinate was the percentage of genes, and the right side is the number of genes.

**Figure 7:**
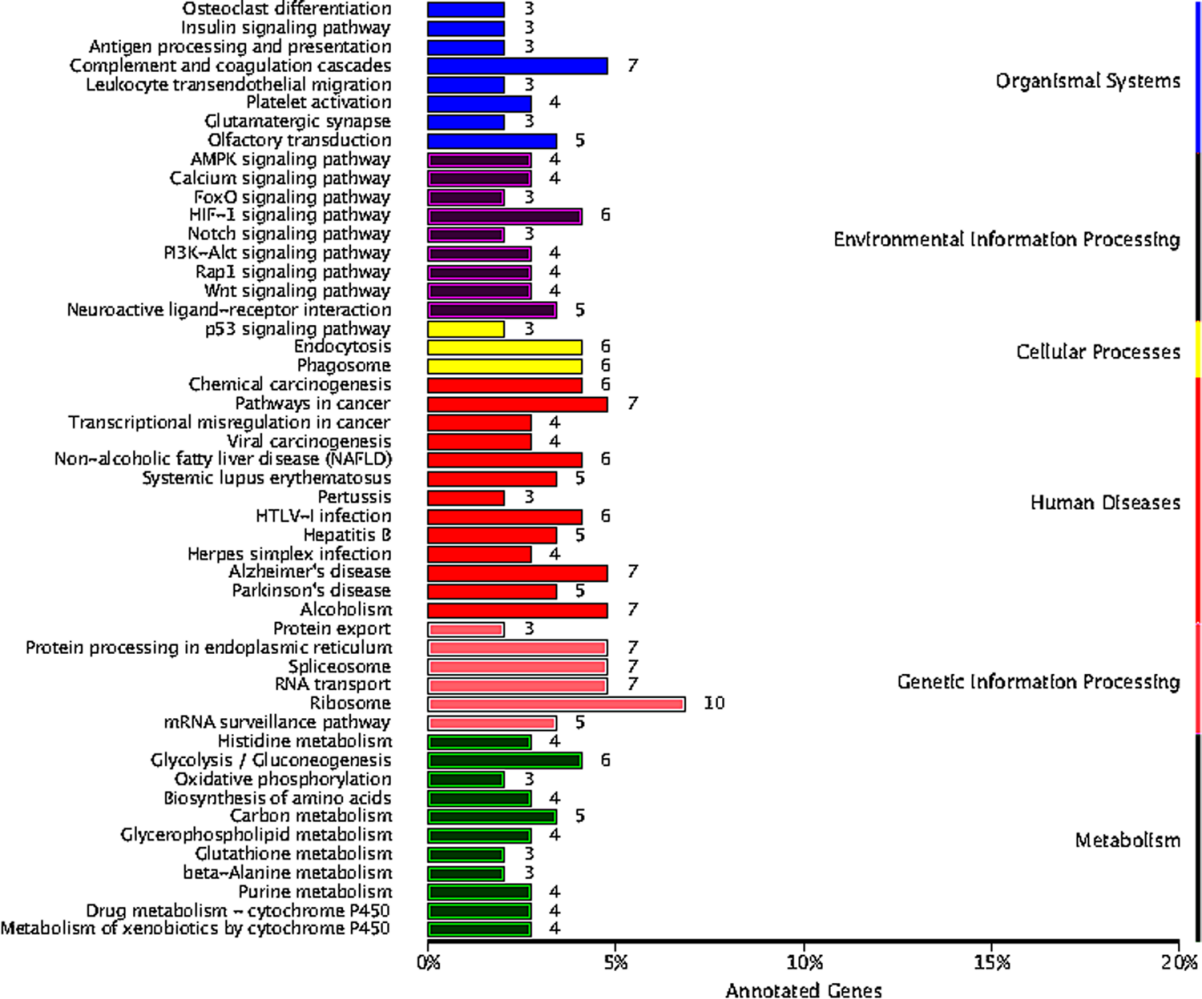
KEGG classification of target genes of DElncRNAs in rats. The abscissa is the annotated genes, the ordinate was pathway term.

The DAVID software was used to predict functions of DElncRNA targets. GO (Gene Ontology) analysis included 19 cell component (CC) terms, 21 molecular function (MF) terms and 22 biological process (BP) terms. Among them, the cell part, membrane part; the binding, catalytic activity, signal transduction activity and biological regulation, metabolic process, response to stimulation had high percentages of genes (Fig. 6). GO results showed that regulation of nucleic acid acid binding, cold stimulation response, regulation of cytokines, regulation of protein complex stability, regulation of protein ubiquitination, metabolic processes, multicellular biological processes and the like. In order to clarify the specific signal pathways affected by the targets, the KEGG (Kyoto Encyclopedia of Genes and Genomes) analysis was conducted. As shown in Fig. 7, the target genes were divided into 50 KEGG pathways. The PI3K-Akt signaling pathway, insulin signaling pathway, T cell receptor signaling pathway, pathways in cancer, fatty acid metabolism, HIF-1 signaling pathway, glucose metabolism and lipid metabolism were mainly involved in the regulation of liver in cold stress rats. Results suggested that these DElncRNAs targets may play crucial roles in energy metabolism, immunity, growth and development, proliferation and apoptosis.

To compare the functions of targets predicted in different ways, GO and KEGG analyses were performed on cis and trans targets respectively from DElncRNAs. GO classification of cis and trans targets from up and down-regulated lncRNAs respectively showed in Supplementary Figs. S1–S4. KEGG classification of cis and trans targets from up and down-regulated lncRNAs showed in Supplementary Figs. S5–S8. The key pathways and functions in red both appear in up- and down-regulate lncRNAs. It indicated that targets were mainly involved in metabolic processes, responses to stimuli, multicellular biological processes, immune system processes, reproductive processes, transport activity, molecular function regulation, nucleic acid binding transcription factor activity, fatty acid metabolism, pathways in cancer and the PI3K-Akt signaling pathway under cold stress.

### Construction of co-expression network to reveal hub lncRNAs

**Figure 8:**
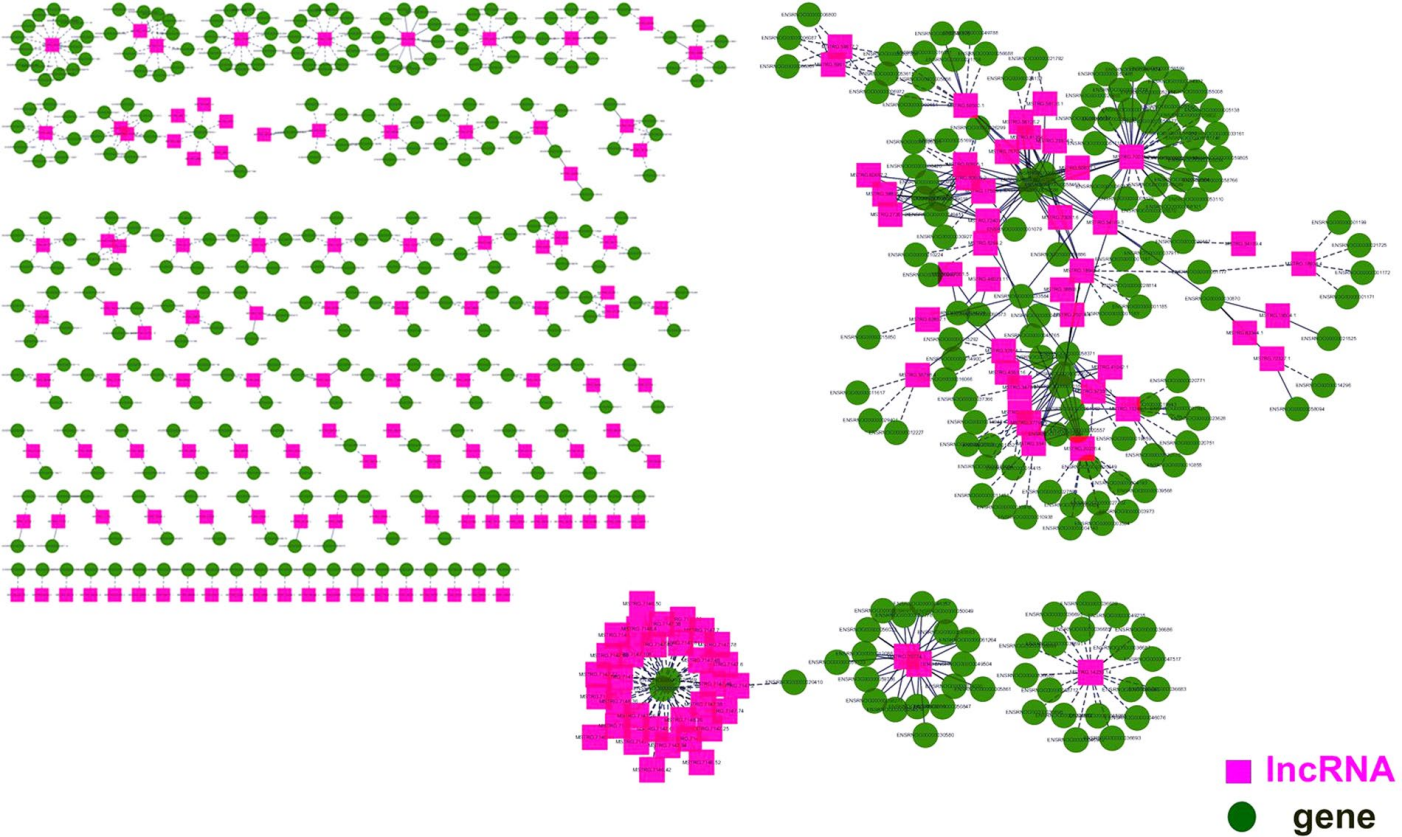
Differential lncRNA interacts with target genes. lncRNAs and coding transcripts were presented as squares and circles, respectively.

**Figure 9:**
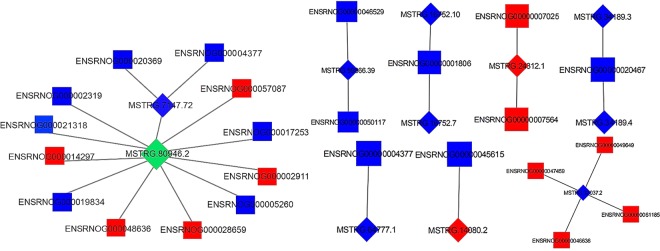
The co-expression network of DElncRNAs and DEmRNAs. lncRNAs and mRNAs were presented as diamonds and squares, respectively. And green and blue indicated down-regulate, red indicated up-regulate.

First, a interaction network of lncRNAs and their targets was constructed using limma platform (Fig. 8). The results showed that a total of 723 interactions were identified among 273 DElncRNA and 415 target genes. There were 517 positive interactions and 206 negative interactions among pairs within the network, and most lncRNA-gene pairs were positively correlated. In addition, one lncRNA can be associated with 1 to 35 mRNAs, and one mRNA can be associated with 1 to 30 lncRNAs. To reveal the most significant hub DElncRNA (P < 0.01), we extracted the junctions between DElncRNA and DEmRNA to reconstitute a co-expression network. As shown in Fig. 9, the two lncRNAs MSTRG.80946.2 and MSTRG.7147.72 were in the center of the network. Compared with MSTRG.7147.72, the MSTRG.80946.2 had higher node degrees and more interactive pairs. This suggested that MSTRG.80946.2 may be a key functional lncRNA in rats liver under cold stress.

**Figure 10:**
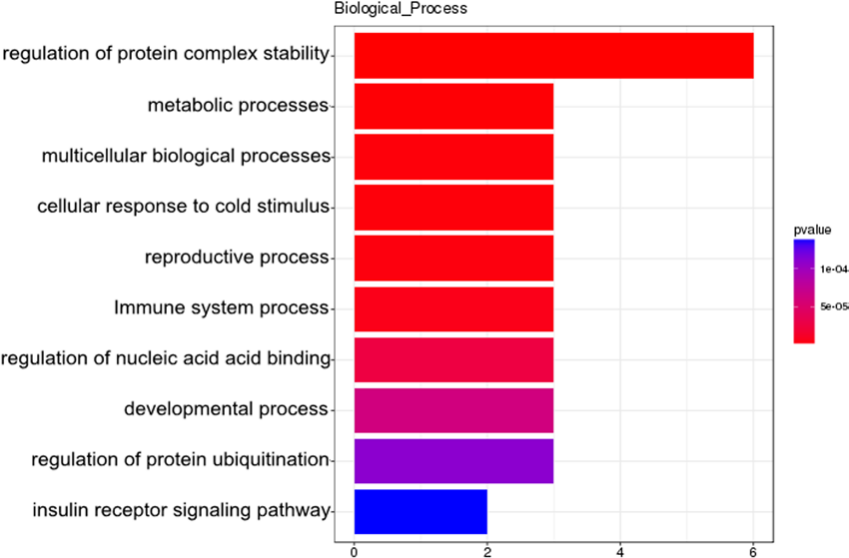
GO classification of co-expressing mRNAs of lncRNA MSTRG.80946.2.

**Figure 11:**
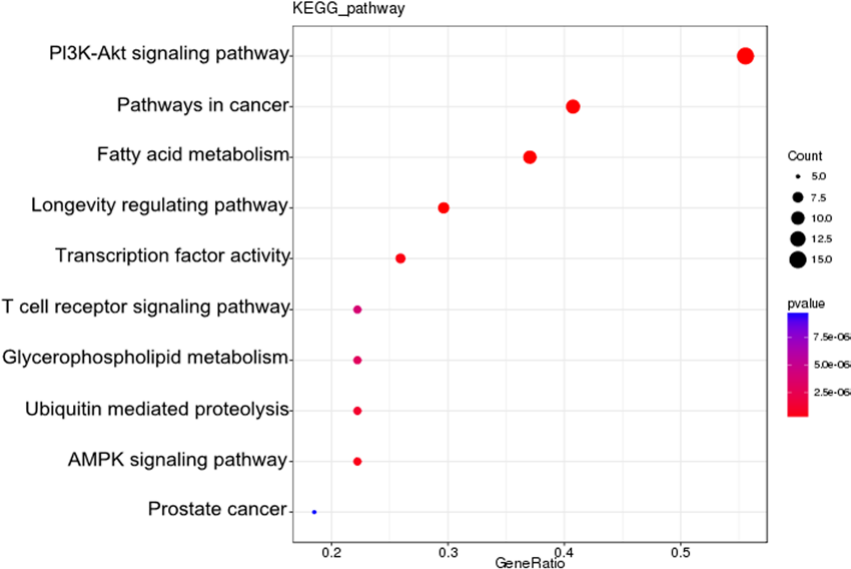
KEGG enrichment analysis of co-expressing mRNAs of lncRNA MSTRG.80946.2.

In order to understand clearer the functions of hub MSTRG.80946.2, the functional analyses were performed on its co-expressing DEmRNAs. GO analysis revealed the top three significantly enriched in “Biological Processes” (BP), including regulation of protein complex stability, metabolic processes and multicellular biological processes (Fig. 10). In addition, the top ten pathways of genes were listed as the most significant p-values. As shown in Fig. 11, KEGG enrichment analysis showed that the top three pathways enriched with most targets were pathways in cancer, the PI3K-Akt signaling pathway and fatty acid metabolism. They respectively included mRNA *TSPY1*, *ACP1*, *Tsn, Hsp90ab1*and so on. Pathways in cancer is closely related to immunity, cell proliferation and apoptosis. PI3K-Akt signaling pathway involves many biological processes, such as protein synthesis, glycolysis, and apoptosis. Studies have reported that lncRNA TUG1 (taurine up-regulated1) inhibits mice apoptosis and thus has a protective effect on cold-induced liver injury^25^. *Translin* was a regulator that responds to metabolic changes^26^. Metabolic status has a major impact on the regulation of biological rhythms. *Hsp90ab1* was an ATP-dependent highly conserved molecular chaperone. It interacted with some epidermal growth factor receptor (EGFR), human epidermal growth factor receptor-2 (HER2), which played an important role in cancer pathway and participates in various pathophysiological processes of cells^24^. *ACP1* was a marker enzyme for lysosomes. As an organelle for digestive function in cells, it contained a large amount of acidic hydrolase, which played an important role in the metabolism of substances inside and outside the cell^27^. These results showed that lncRNA targets were prominent in metabolic disorders and cancer pathways under cold stress.

### Quantitative analysis verified sequencing accuracy

**Figure 12:**
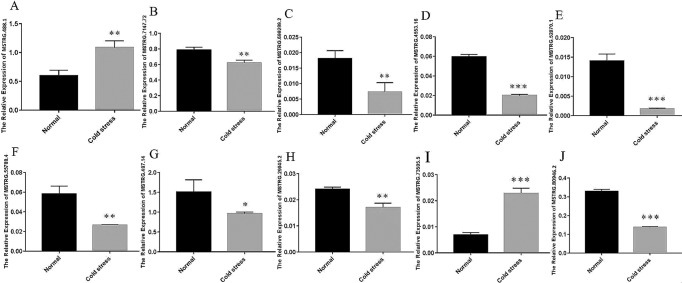
qRT-PCR validation of high throughput sequencing. Validation of 10 selected DElncRNAs. T-test p-values  < 0.05 are considered to be significantly different, “*” represents a p-value  < 0.05 and “**” represents a p-value  < 0.01.

We selected 10 significantly DElncRNAs (MSTRG.488.1, MSTRG.73505.5, MSTRG.7147.72, MSTRG.69299.2, MSTRG.4553.16, MSTRG.52070.1, MSTRG.29045.2, MSTRG.55788.4, MSTRG.487.14 and MSTRG.80946.2) from cold-stressed rat livers to verify the accuracy of sequencing results by qRT-PCR (Fig. 12). The results illustrated that the relative expressed changes of lncRNAs in conformity with high-throughput sequencing results, indicating that expressed assessment and identification of lncRNAs were persuasive. In all DElncRNAs, MSTRG.80946.2 was the most significantly DE (P < 0.001) under cold stress. Therefore, further functional verification of this key lncRNA was performed.

### Characteristic analysis of the key lncRNA

**Figure 13:**
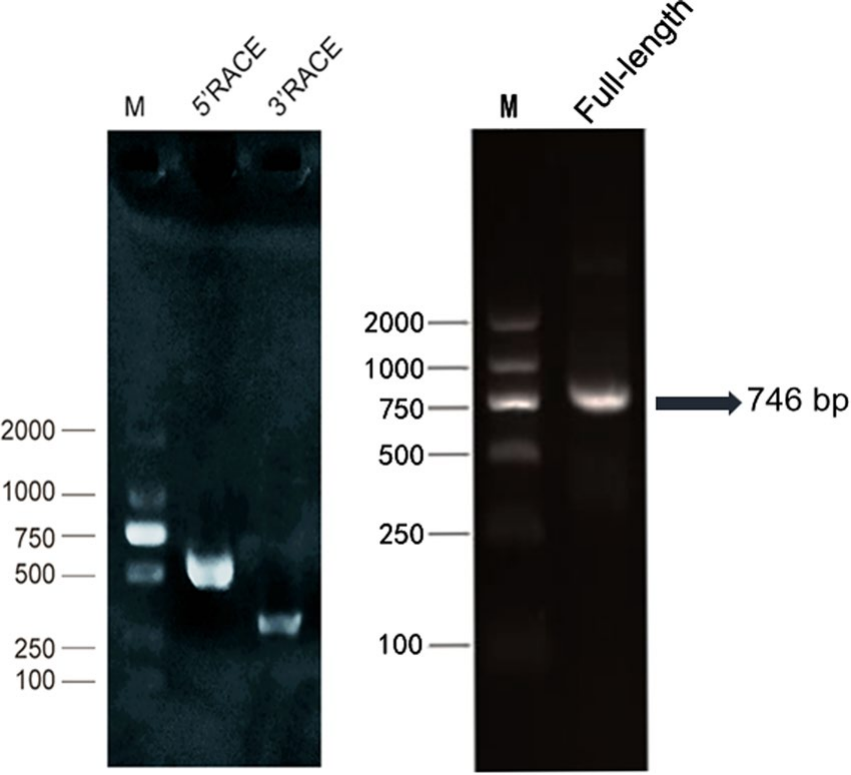
The agarose gel electrophoresis map of RACE product. M: DL2000 DNA Marker. 5′RAEC: 583 bp (include link sequence); 3′RACE: 383 bp (include link sequence).

**Figure 14:**
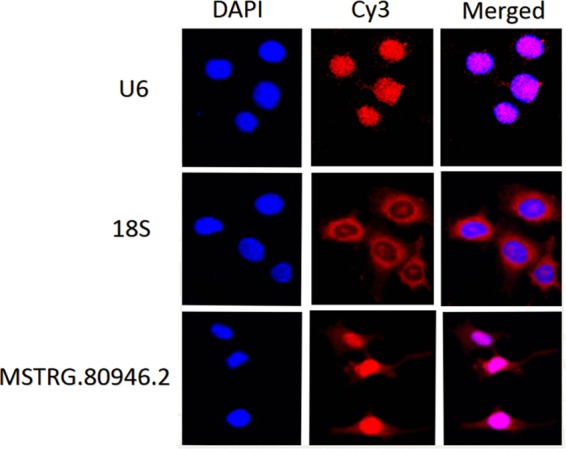
The subcellular positioning of MSTRG.80946.2 in BRL cell.

We compared MSTRG.80946.2 with the known rat sequence in NONCODEv5 database, which was closest to NONNATT021477.2 in length and chromosomal location with 99% homology. So far, there was little information about NONNAT021477.2, only its sequence length was 712 bp and located in chr4. The full length of MSTRG.80946.2 was amplified by RACE (rapid amplification of the cDNA ends). As shown in Fig. 13, the length of the 5′ RACE was 583 bp, and the length of the 3′ RACE was 383 bp. After the linker sequence was removed, the full-length was spliced to 746 bp. The RACE products were subjected to agarose gel electrophoresis. The results showed a distinct band at 746 bp. Next, we performed BLAST alignment using the full length of MSTRG.80946.2 and the known rat sequence from NCBI Genebank. It was found that its sequence was inversely complementary to the sequence of acid phosphatase 1 (*ACP1*) (Supplementary Fig. S9). In a comparison with the co-expression network (Fig. 9), *ACP1* may be a target gene of MSTRG.80946.2 by Cytoscape.3.60. This illustrated that our analysis was aligned with the sequencing results. Regulating the expression of adjacent genes is one way in which lncRNAs act^28^. Thus, we hypothesized that MSTRG.80946.2 may play a part in rats liver under cold stress by regulating *ACP1* expression. Then the subcellular localization of MSTRG.80946.2 in BRL (Rat liver cell) was verified by fluorescence *in situ* hybridization (FISH). The Fig. 14 showed that MSTRG.80946.2 was mostly expressed in the nucleus, and expressed at a low level in the cytoplasm.

### Validation of key lncRNA and its targets in vitro

**Figure 15:**
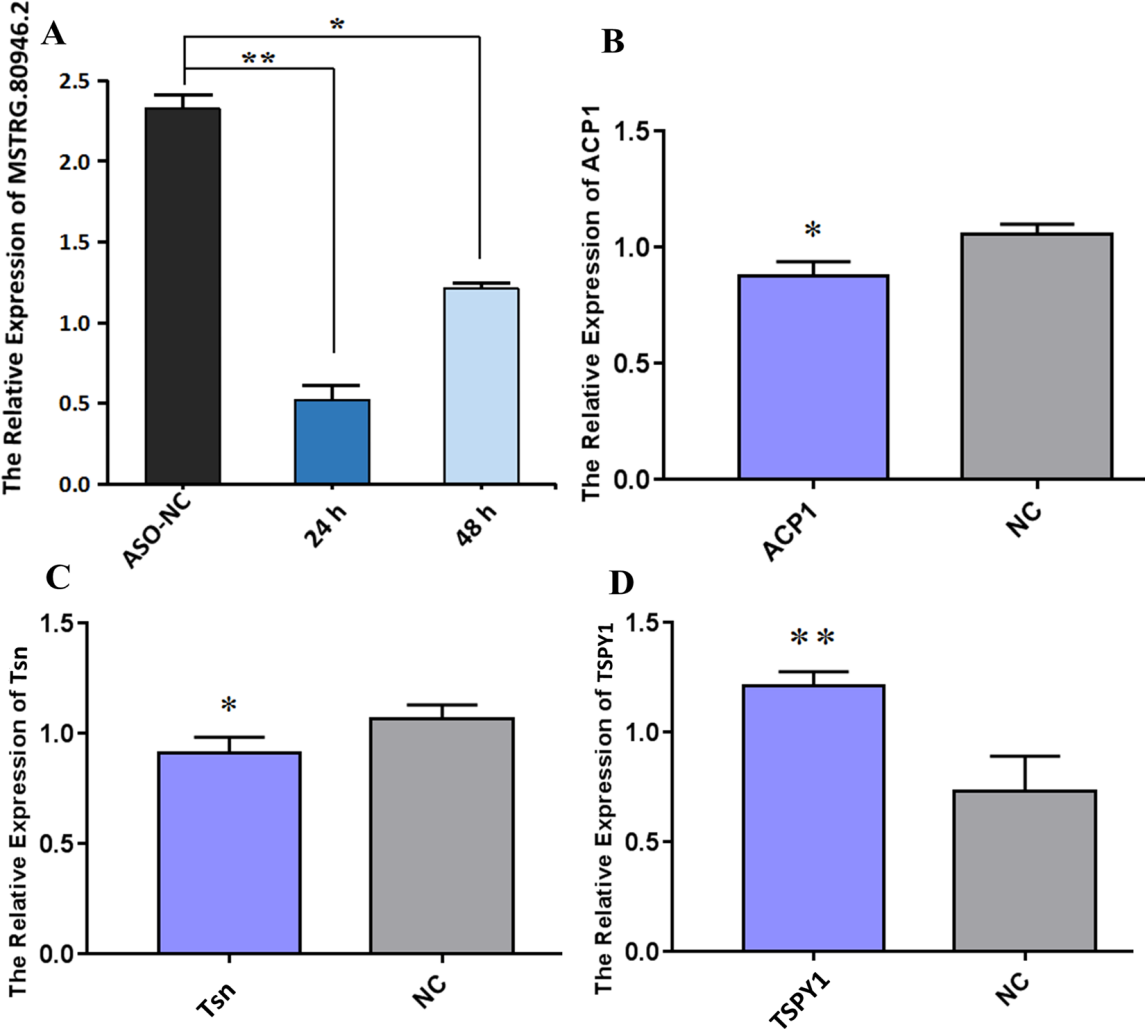
qRT-PCR verification of relative expression after ASO-MSTRG.80946.2 silencing. (**A**) The relative expression of MSTRG.80946.2. (**B**) The relative expression of *ACP1*. (**C**) The relative expression of *Tsn*. (**D**) The relative expression of *TSPY1*. *P < 0.05, **P < 0.01.

In order to verify the relationship between MSTRG.80946.2 and its targets, we transfected ASO targeting MSTRG.80946.2 into BRL cells. The Fig. 15(A) confirmed the silencing effect of MSTRG.80946.2 compared to control ASO-NC, and interference efficiency at 24 h was higher than 48 h. After silencing of lncRNA MSTRG.80946.2, the adjacent gene *ACP1* and *Tsn* were significantly down-regulated (Fig. 15B,C). The *TSPY1* expression was extremely up-regulated (Fig. 15D). The results showed that lncRNA MSTRG.80946.2 did regulate the protein-coding genes *ACP1, TSPY1* and *Tsn* to play an important part in rats liver under cold stress.

## Discussion

Cold stimulation is the most common stressor in cold regions. The slow growth of animals, poor disease resistance, and even death due to cold are important factors restricting the development of animal husbandry in cold regions. As high-throughput sequencing technology continues to improve, cold stress-related lncRNAs have been found in fish and plants^29–31^. However, few researches have been done on mammalian cold stress-related lncRNAs. We previously established a cold stress rat model and confirmed that the liver is a key target organ for cold stress injury. This study used HiSeq. 2500 high-throughput sequencing technology to construct differential expression profiles of cold stress-related lncRNAs in rats liver. 6,025 lncRNAs and 273 DElncRNAs were identified. GO and KEGG analysis of DElncRNA targets indicated that targets were mainly related to PI3K-Akt signaling pathway, positive regulation of cell division, pathways in cancer, fatty acid metabolism, multicellular biological processes, immune system processes, reproductive processes, transport activity, molecular function regulation and nucleic acid binding transcription factor activity. In addition, a co-expression sub-network containing 11 DElncRNAs and 24 DEmRNAs was reconstructed to reveal the underlying mechanisms of cold stress. Functional analyses were further performed on target genes of hub lncRNA MSTRG.80946.2 in sub-network. KEGG enrichment analysis showed that the top three pathways enriched with most targets were pathways in cancer, the PI3K-Akt signaling pathway and fatty acid metabolism. Liu *et al*. found that brain damage caused by cold stress can be prevented by inhibiting TRPV1 (transient receptor potential vanilloid subtype 1) and the PI3K/Akt inflammatory pathways^32^. We also found that some lncRNAs regulated the expression of their target genes through cis mechanism. For example, lncRNA MSTRG.7147.72 (1910 bp, chr 1) was down-regulated under cold stress, and can target two cis genes, *Igf2* and *Ins2*. Many of the identified lncRNAs were not found in public data, and there was little information to describe the functional annotations of co-expressed genes. For example, MSTRG.62962.1, ENSRNOG00000042133 and ENSRNOG00000059588 showed strong co-expression, but annotation of these target genes requires further investigation.

MSTRG.80946.2 was the hub lncRNA in the net. Currently, there were few reports on this lncRNA. In this study, it was identified as a known lncRNA with 99% homology to NONNATT021477.2, and had 10 target gene pairs (Fig. 9), including *TSPY1*, *ACP1*, *Tsn*, *Il2rb*, *SZT2*, *Lpin1*, *EPAS1*, *Hsp90ab1*, *Alb* and *Ccdc107*. Testis-specific protein Y-encoded (*TSPY*) is expressed in sperm cells of adult animal testes^33^. *TSPY1* is related to male testis and fertility. *TSPY* and its homologous *TSPX* act as protooncogenes and tumor suppressor genes, respectively. They have opposite effects on cell proliferation and degradation of viral HBx (Hepatitis B virus X protein) oncoprotein^34^. Recently, human *TSPY* has been reported to inhibit USP7 (ubiquitin-specific peptidase 7) -mediated p53 function and promote spermatogonia proliferation A^35^. The deletion of *Il2rb* causes mice to develop immune disease and NK cell dysfunction, including severe autoimmunity^36^. Loss of function of *SZT2* leads to over-activation of mTORC1 signaling, due to amino acid deficiency^37^. There is increasing evidence that *SZT2* is associated with neurological diseases such as growth retardation and epilepsy. *SDC* belongs to the family of HSPG (heparan sulfate proteoglycans)^38^. Giuseppina *et al*. found that *SDC4* may regulate lipid homeostasis and play a key role in human health and longevity^39^. Studies have shown that translin/trax RNase complex can degrade microRNA, thereby regulating energy metabolism^40^. Lack of *Lpin1* can lead to severe metabolic homeostasis, such as fatty liver and cardiovascular disease. It regulates cellular triacylglycerol levels and liposomes in cellular signaling pathways^41^. Lipoproteins as a target for inflammatory diseases or metabolic therapies require further investigation. Herui *et al*. established a mouse model to confirm that the *EPAS1* mutation is a causative gene for somatostatin^42^. Studying the HIF-2α function associated with *EPAS1* helps to discover the role of tumors. HSP (Heat Shock Protein) can form a multi-protein chaperone complex involved in the proliferation of animal cells and the folding of apoptotic substrates^43^. Inhibition of *Hsp90* leads to ubiquitination of the proteasome pathway^44^. *Hsp90ab1* is one of its isoforms. *Ccdc107* is one of the family of helical coil domains (Ccdc). Ccdc has many important biological functions and can regulate various biological behaviors such as invasion and metastasis of malignant tumor cells^45^. It has been confirmed that Ccdc protein is abnormally expressed in prostate cancer, breast cancer and so on^46^. And it has a direct link with tumor cell migration and invasion. These target gene reports above suggested that lncRNA may affect the physiological processes of energy metabolism, reproductive performance, immunity, apoptosis and proliferation in rats under cold stress. It is well known that the effects of low temperature environments on the energy metabolism of living organisms are considerable large^47^. Cold stress can damage macromolecules such as proteins, nucleic acids and lipids in body cells. These molecular deletions lead to metabolic disorders and changes in redox potential^48^.

In further study, lncRNA MSTRG.80946.2, which is closely related to cold stress and has a significant expression level, was selected for verify in cells to elucidate its mechanism of action. We found that MSTRG.80946.2 is 99% homologous to NONNATT021477.2 from NONCODEv5 database. MSTRG.80946.2 is mainly expressed in the nucleus of BRL by FISH (fluorescence *in situ* hybridization), so subsequent gene silencing experiments prefer ASO technology. The full length of MSTRG.80946.2 was amplified by RACE. It was found to be an antisense lncRNA of rat *ACP1* compared with NCBI database. ACP1 is a lysosomal marker enzyme that is involved not only in intracellular digestion and endocytosis of phagocytic cells, but also in important life activities, such as nucleic acid and protein metabolism, immune regulation and signal transduction^49^. Wang *et al*. found that *ACP* has strong activity in fish liver and is widely involved in the energy metabolism^50^. Interestingly, *ACP1* was one of DEmRNAs of MSTRG.80946.2 in the co-expression network. Therefore, we further verified the relationship between MSTRG.80946.2 and its targets in BRL cells. It was confirmed that MSTRG.80946.2 did regulate *ACP1*, *TSPY1* and *Tsn* expression in rats under cold stress.

In conclusion, this study was the first to systematically identify cold stressed-associated lncRNAs in rats liver and construct lncRNAs DE profiles. lncRNAs played crucial roles in energy metabolism, reproductive performance, growth and development and immunity in rats by regulating mRNAs under cold stress. The MSTRG.80946.2 was verified by network and experiments to be a cold responsive key lncRNA, regulating protein-coding genes *ACP1*, *TSPY1* and *Tsn*. However, the detailed mechanism of lncRNAs under cold stress still requires further experimental verification.

## Materials and Methods

### Ethics statement

This research strictly followed the principles of animal use in the China Laboratory Animal Science Association. And the study obtained the approval of the Animal Ethics Committee of Heilongjiang Bayi Agricultural University. In order to reduce animal suffering, all surgery was performed under sodium anesthesia with pentobarbital.

### Samples collection, library preparation and high-throughput sequencing

Our team established an animal cold stress model early on and confirmed that liver is a key target organ for cold stress injury. Six 12 weeks old SPF Wistar male rats were equally divided into two groups, using artificial intelligence climate chamber for cold stress group (4 ± 0.05) °C and normal control group (24 ± 0.05) °C kept for 12 h. The relative humidity was 45 ± 0.10%, and diets or water and other conditions were treated equally. The next day, liver samples were collected from anesthetized rats to extract total RNA. According to manufacturer’s description to construct cDNA libraries using SMART Kit (Clontech, USA)^51^. The HiSeq. 2500 plat was used to perform sequencing. Processing raw data for quality control and got Q30 and GC sizes of clean data. High throughput sequencing was performed by Biomarker Technologies Co., Ltd. (Beijing, China).

### Bioinformatics identification of lncRNAs

Screening transcripts with length ≥200 bp and exon number ≥2. The coverage of each transcript was calculate by cufflinks software, and selected the transcript with reads minimum coverage ≥3. The lncRNAs spliced by two splicing softwares and screened out to obtain new predicted lncRNAs set. We used cuffcompare information to screen different types of lncRNAs such as lincRNA, intronic lncRNA, and anti-sense lncRNA. Four methods for screening transcript coding potential by CNCI, PhyloCSF analysis, CPC and pfam protein domain analysis^52–54^.

### Analysis of DElncRNAs

The expression level of lncRNAs was estimated by FPKM (fragments per kilobase of transcript per million fragments mapped). In the process of differential expression of lncRNAs, Fold Change ≥2 and FDR < 0.01 were used as screening criteria^55^. Hierarchical clustering analysis of DElncRNAs.The cis target genes were predicted to analyze genes in range of 10 kb up- and down-stream of lncRNA on same chromosome. The prediction of trans target genes based on possible co-expression relationship between lncRNA and mRNA^56^. GO and KEGG analysis of DElncRNA target genes by DAVID (https://david.ncifcrf.gov/).

### lncRNA-mRNA networks construction

DElncRNA-mRNA interaction network was created for Cytoscape visualization^57^. And selected key node DElncRNA and DEmRNA to construct co-expression regulatory network. Furthermore, screening for the hub lncRNA via this network.

### Verification of sequencing results by qRT-PCR experiment

To further verify the sequencing results, we selected 10 DElncRNAs randomly to qRT-PCR. Follow the 2 × Taq SYBR Green qPCR Mix Kit, and the reaction system was 25 μl. β-actin as an internal reference primer. Primers were designed using Olige6 online software. Primers were synthesized by Sangon (Shanghai, China). Forward and reverse primer sequences: MSTRG.80946.2: F: 5′-GCCATGCTCACACTCCAGTT-3′ R: 5′-GGAACGAGCAACTGTGGAAC-3′; MSTRG.488.1: F: 5′-AGAGCGCCAGCTATCCTGA-3′ R: 5′-AACTCTGGTGGAGGTCCGTAG-3′; MSTRG.7147.72: F: 5′-CTTGGTGGTCTGCTGTCTGG-3′ R: 5′-CCATAGGCTTCATTGCGTCTT-3′; MSTRG.69299.2: F: 5′-TTCTTGGCACATAGCATGGA-3′ R: 5′-TCCTGGACTGGCATTGACTT-3′; MSTRG.4553.16: F: 5′-CTTGCTGCCTTCCAGCCTA-3′ R: 5′-CATGCGTGTGTGTGTGTGTG-3′; MSTRG.52070.1: F: 5′-CACGCAAGTCTCTGACACGA-3′ R: 5′-TCTTACACCGCGTCTGCACT-3′; MSTRG.29045.2: F: 5′-TCACACAGTTCACGCAAGGA-3′ R: 5′-GGCCACACAGATTGGACAGT-3′; MSTRG.55788.4: F: 5′-AGACGTGCCAAGGACACAGA-3′ R: 5′-TCAGGACCTCTGGAAGAGCA-3′; MSTRG.487.14: F: 5′-CGGCCAGCGAGAAACGAAAC-3′ R: 5′-TCCTGTGTGTCCCGCCTTTC-3′; MSTRG.73505.5: F: 5′-CTGTGTGCAATGGCCTATCTC-3′ R: 5′-TGCTGGAATGAATGCTGGAT-3′. Results data are expressed as mean ± standard deviation. ANOVA (One-way analysis of variance) was used to check the significance of the mean using Graphpad Prism 7.0.

### Rapid amplification of the cDNA ends

To identify the full length sequence of lncRNA MSTRG.80946.2, RACE experiments were performed using the SMARTerTM RACE cDNA Amplification Kit (Clontech, USA) according to the manufacturer’s instructions. The general procedure of 5′ RACE and 3′ RACE were as follows: synthesis of first strand cDNA, amplification of target cDNA, cloning and amplification of RACE product, screening and sequencing. The product was subjected to agarose gel electrophoresis. The specific primers sequences are as follows: 3′ RACE: TGCTCACACTCCAGTTAGACCAG; 5′ RACE: CAGACATTTGAGGTGTGGCCT.

### Cell culture

BRL cells (normal rat hepatocytes) were purchased from the Chinese Academy of Sciences Cell Resource Library (Beijing, China). The cell culture medium were10% FBS, 10 ml fetal bovine serum (gibco, USA) added 90 ml DMEM high glucose medium (gibco, USA), and penicillin-streptomycin solution 0.1%. The cells were cultured in 37 °C, 5% CO_2_ incubator (Binder, Germany), updated the solution after 24 h.

### Fluorescence in situ hybridization

FISH was performed according to fluorescence *in situ* hybridization kit (RiboBio, Guangzhou, China). BRL cells were washed with PBS and fixed in 4% paraformaldehyde for 15 min. Then, the permeate of 0.3% Triton X-100 was added to stand for 8 min, washed twice with PBS, and kept at 37 °C for 30 min. Anti-MSTRG.80946.2, anti-U6 or anti-18S probes were hybridized overnight at 37 °C. The next day, DAPI counterstained the cells and imaged using CLSM (confocal laser scanning microscope) (Leica, Germany).

### *In vitro* lncRNA silencing assay

Specific ASO interference sequence targeting lncRNA MSTRG.80946.2 by Ribobio Biotechnology (Guangzhou, China). The ASO plasmids were transfected into BRL cells at 200 nmol for 24 h, and then subjected to qRT-PCR. The ASO sequence are: ASO-MSTRG.80946.2: 5′-TTAACTTCACCAACCTGTTG-3′, ASO-NC: 5′-TTAAATGGAAGGCTGCCATG-3′. Transfection was performed using Lipofectamine RNAiMAX (Thermo Scientific, USA).

## Supplementary information

Supplementary Information.
