# Supplementary material for: Identification, functional prediction, and key lncRNA verification of cold stress-related lncRNAs in rats liver
Source: Sci Rep. 2020 Jan 16;10:521. doi: 10.1038/s41598-020-57451-7 (PMC6965121; doi:10.1038/s41598-020-57451-7)
Supplement: Supplementary file 1 — Supplementary Information. [file 41598_2020_57451_MOESM1_ESM.doc]

**Identification, functional prediction, and key lncRNA verification of cold stress-related lncRNAs in rats liver**

Hong Ji[[1]](#footnote-2), Chunyang Niu^1^, Xuelong Zhan^1^, Jing Xu^1^, Shuai Lian^1^, Bin Xu^1^, Jingru Guo^1^, Li Zhen^1^, Huanmin Yang^1^, Shize Li^1^ & Li Ma^1^


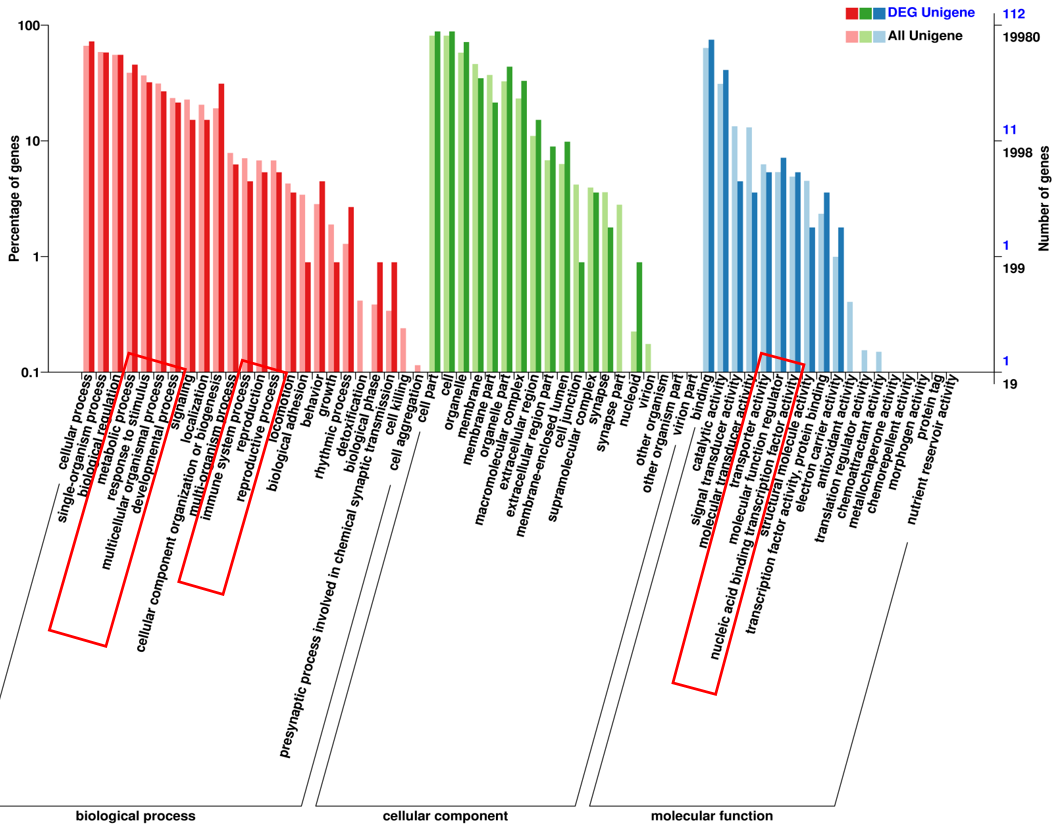


**Supplementary Fig. S1.** GO analysis of target genes down-regulated lncRNAs by cis.


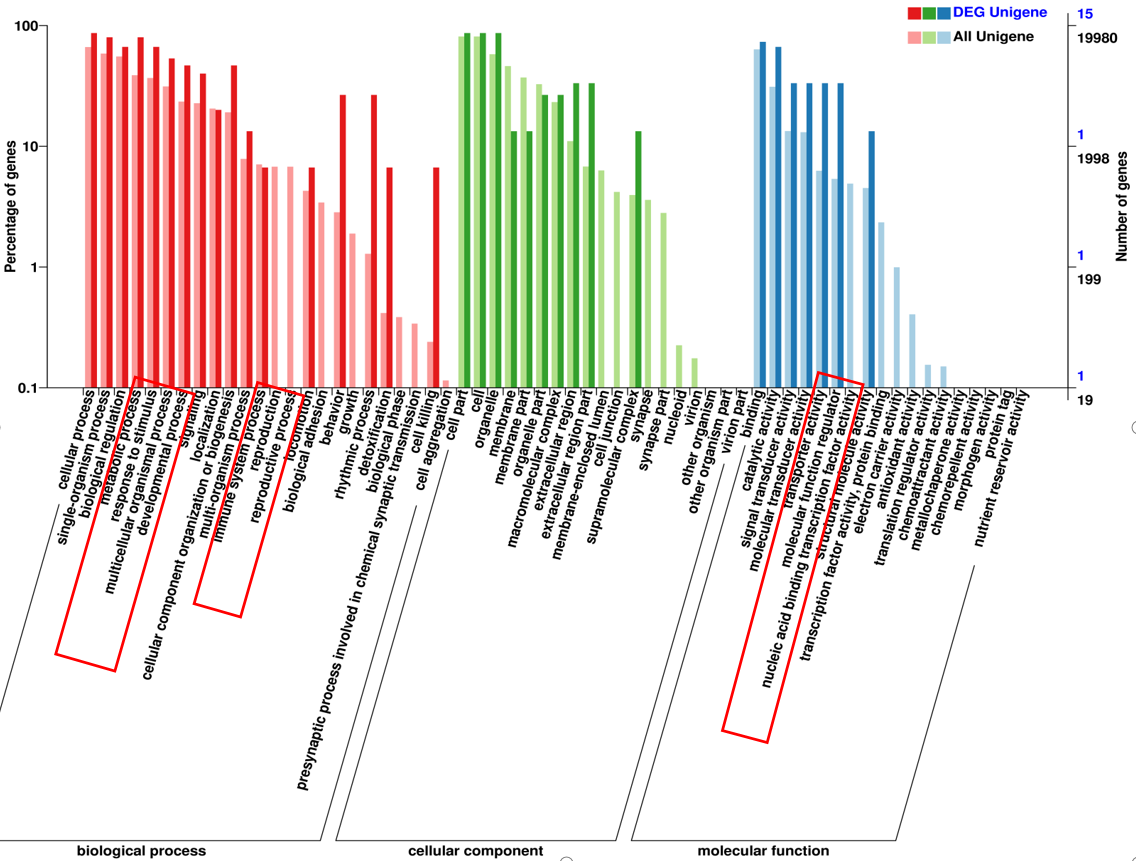


**Supplementary Fig. S2.** GO analysis of target genes down-regulated lncRNAs by trans.


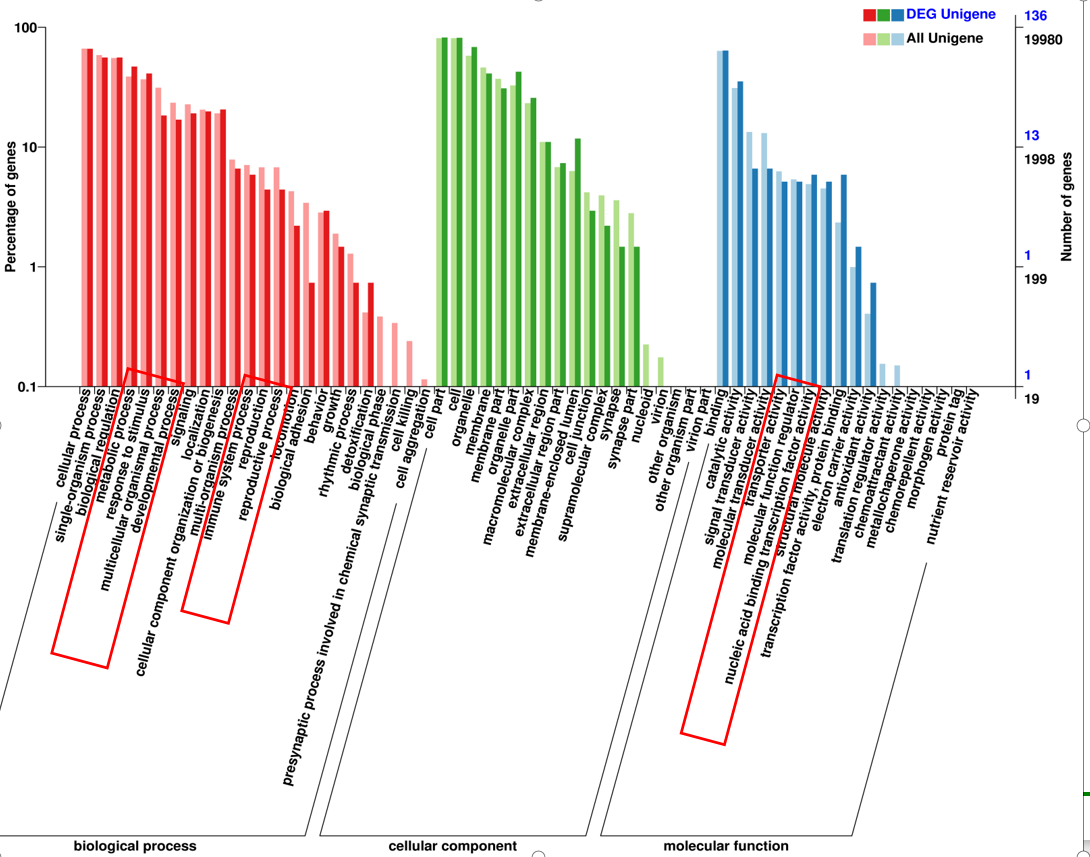


**Supplementary Fig. S3.** GO analysis of target genes up-regulated lncRNAs by cis.


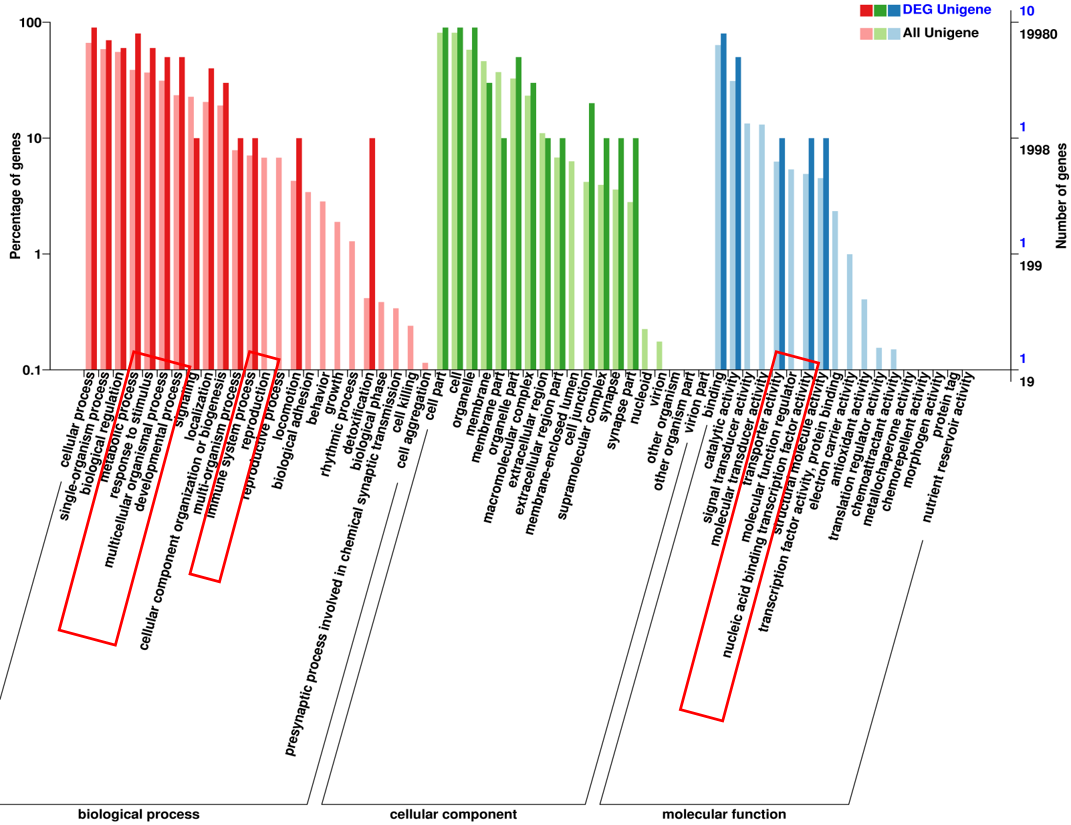


**Supplementary Fig. S4.** GO analysis of target genes up-regulated lncRNAs by trans.


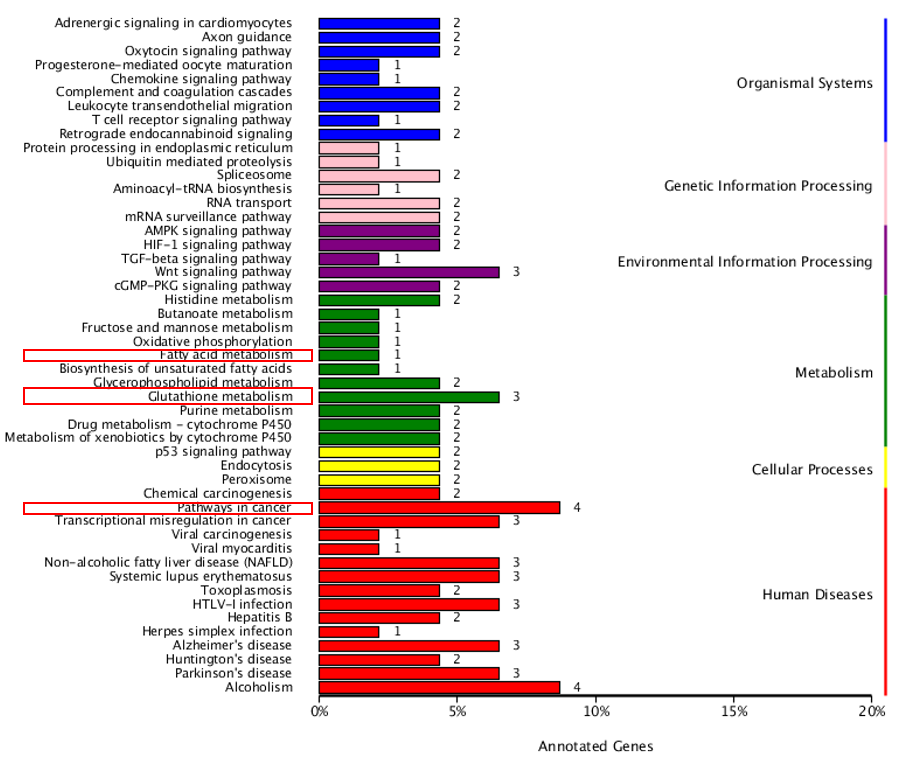


**Supplementary Fig. S5.** KEGG analysis of target genes down-regulated lncRNAs by cis.


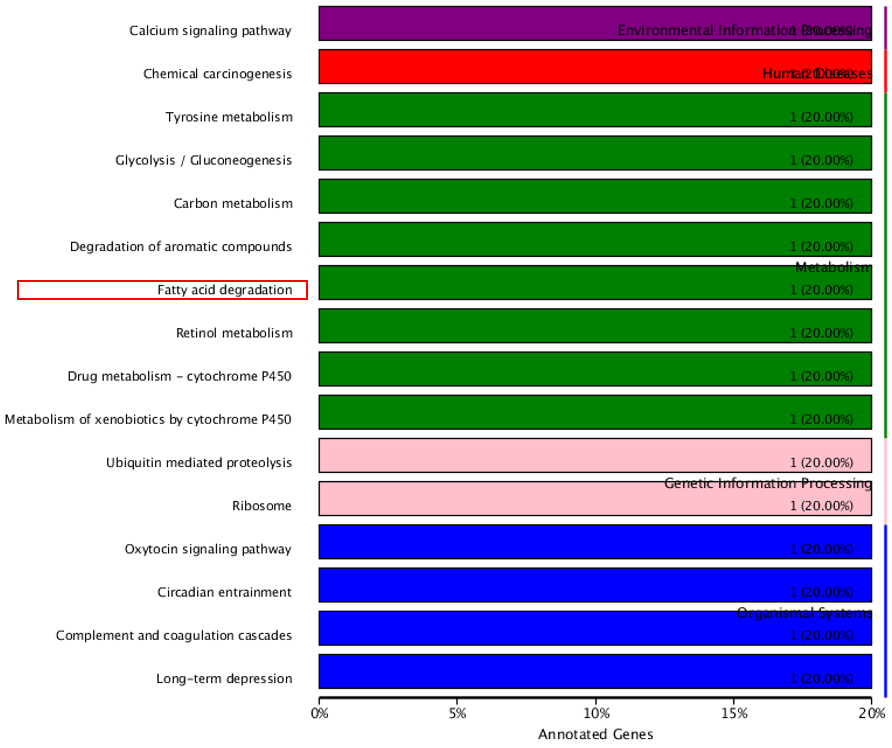


.

**Supplementary Fig. S6.** KEGG analysis of target genes down-regulated lncRNAs by trans.


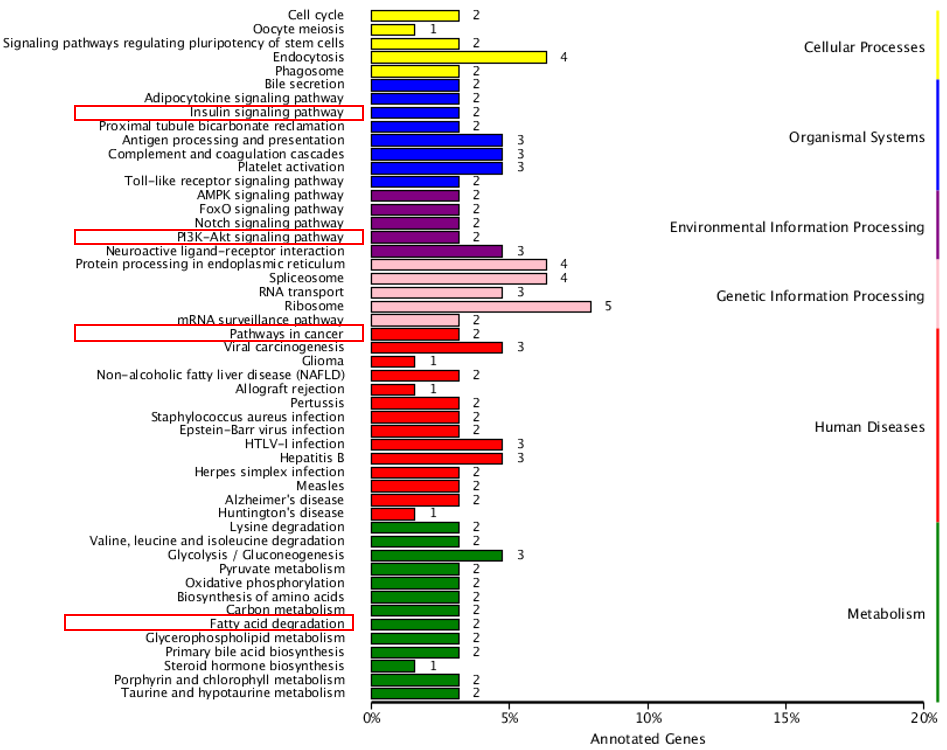
.

**Supplementary Fig. S7.** KEGG analysis of target genes up-regulated lncRNAs by cis.


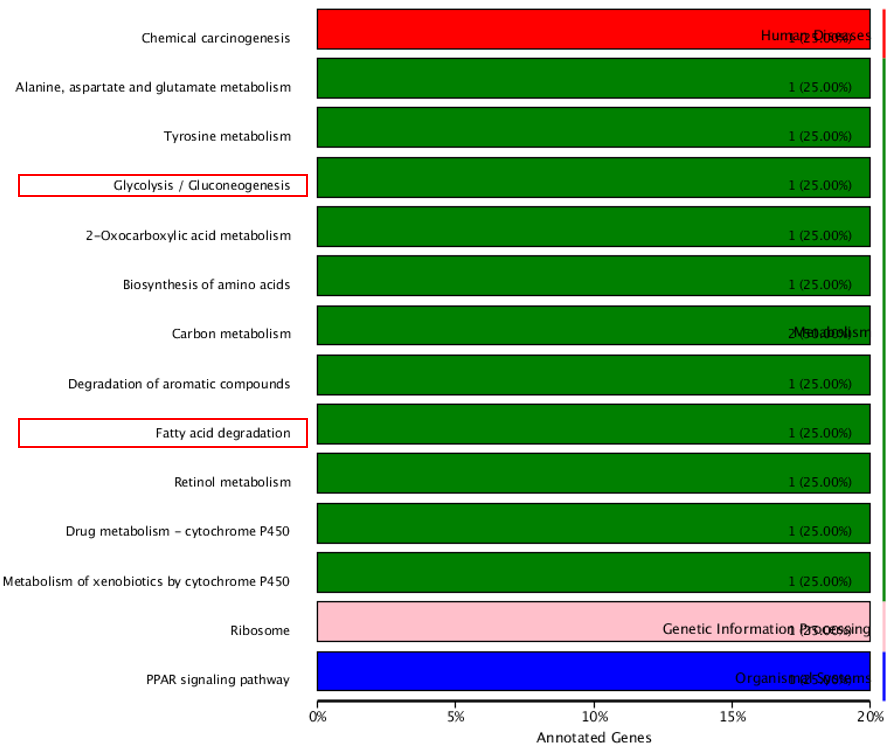


**Supplementary Fig. S8.** KEGG analysis of target genes up-regulated lncRNAs by trans.


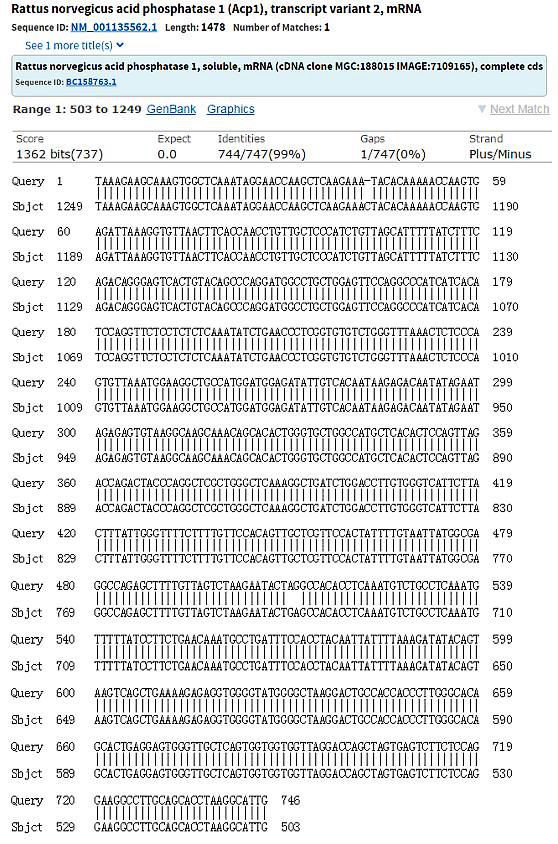


**Supplementary Fig. S9.** The BLAST alignment map of lncRNA MSTRG.80946.2.

1. ^1^College of Animal Science and Veterinary Medicine, Heilongjiang Bayi Agricultural University, Daqing 163319, China. Hong Ji and Chunyang Niu contributed equally to this work. Correspondence and requests for materials should be addressed to S. L. (email: byndlsz@163.com) or L. M. (email: 250507411@qq.com) [↑](#footnote-ref-2)
